# Supporting Governance of Economic Development: The PAANEEAC Experience in Central Africa

**DOI:** 10.1177/0169796X17694447

**Published:** 2017-05-22

**Authors:** Geske Dijkstra, Dieudonné Bitondo, Sibout Nooteboom, Reinoud Post, Gwen van Boven

**Affiliations:** Erasmus University Rotterdam, The Netherlands; University of Douala and Secretariat for Environmental Assessment in Central Africa, Cameroun; Erasmus University Rotterdam and The Netherlands Commission for Environmental Assessment; The Netherlands Commission for Environmental Assessment; The Netherlands Commission for Environmental Assessment

**Keywords:** governance, capacity development, Africa, self-organization, environmental impact assessment

## Abstract

This article aims to evaluate PAANEEAC (Projet d’appui au développement des associations nationales pour l’évaluation environnementale en Afrique Centrale), the program to support the development of national associations (NAs) for environmental impact assessment (EIA) in Central Africa. PAANEEAC’s objective is to improve the governance of investment decisions through strengthening capacities for EIA. From the literature explaining the failure of donor-induced capacity development programs, the article deduces conditions for success. The empirical assessment of PAANEEAC is based on document review, extensive interviews, and observations. It concludes that PAANEEAC managed to create platforms in which stakeholders meet with the common objective of improving EIA systems, and that this led to measurable, albeit modest improvements in EIA systems. Furthermore, PAANEEAC met most of the success conditions, which was instrumental for its performance.

## Introduction

It is commonly believed that lack of appropriate institutions and lack of capacities are the main obstacles for development. Hence, donors have invested massively in technical assistance in order to improve institutions and capacities. According to the OECD, about a quarter of all aid, or around US$25 billion annually, has been spent on technical assistance, and most of this has been for capacity development. Yet, “much of this money has had relatively little impact.”^1^ Although it varies per area, with some more success in the area of public financial management, the general finding is that external efforts to improve the capacity of the public sector have not been very successful (IEG, 2008; Kapur, 2001; Smets, 2014). There is an emerging literature on aid for capacity development that attempts to explain the limited success of this aid modality (Andrews, Pritchett, & Woolcock, 2012; Armstrong, 2013; Hyden, 2013).

This article aims to use this literature to explain the results of a program to improve capacities for environmental impact assessment (EIA) in Central Africa, PAANEEAC, the program to support the development of national associations (NAs) for impact assessment in Central Africa (in French: Programme d’Appui au développement des Associations Nationales pour l’Évaluation Environnementale d’Afrique Centrale). EIA is considered to be a key element of the regulatory chain, which runs from strategic government investment decisions to the enforcement of the conditions for the operation of facilities. As a public procedure, it is used in almost every country of the world, to enable governments to take environmental impacts into consideration when giving consent to development projects (e.g., Nooteboom & Teisman, 2003). In many African countries, EIA also encompasses social impacts; in fact, it covers all effects that are not primarily intended by the developer’s design. In addition, EIA aims to establish a transparent, participatory decision-making process on investment projects. Whereas it is widely accepted that many specialist technical questions can only be answered by experts and scientists, perceptions of civil society and citizens may be seen as “lay knowledge” that is relevant as well. EIA links both types of knowledge in decision-making by making all considered alternatives and their perceived impacts transparent and making governments accountable.

PAANEEAC was a capacity development program with a small grants component, financed by the Dutch Ministry of Foreign Affairs and administered through The Netherlands Commission for Environmental Assessment (NCEA). It aimed to support NAs of EIA professionals in Central Africa (Burundi, Cameroon, Congo Brazzaville, Central African Republic, and Rwanda) and their regional secretariat (known after its French acronym SEEAC^2^ (Secretary for the Environmental Assessment in Central Africa)). It has been implemented from 2008 to 2013. The NAs and SEEAC had as joint objective to improve the national EIA systems and to promote regional harmonization of those systems, with a view to good governance, poverty reduction, and sustainable development.

This article aims to answer the question to what extent has PAANEEAC been successful, and how can this success, or lack of success, be explained? The article begins by reviewing the literature on explaining the limited results of donor efforts to improve governance and institutions. From this literature, we derive conditions for success. The next section describes the origin of the PAANEEAC program and the methodology for examining the processes and outcomes of PAANEEAC. We then describe the PAANEEAC activities and examine its outcomes. Finally, we analyze to what extent the PAANEEAC program met the defined success conditions. The conclusion brings the different lines together.

## Aid for Capacity Development

This section aims to explain why so many donor-induced efforts to promote institutional change and capacity development did not succeed, and to analyze under what conditions these efforts can be successful.

One of the most important causes of the limited impact of capacity development projects is the fact that most of this technical assistance is supply driven. External “experts” come with their blueprints and “best practices” and do not take local conditions sufficiently into account. Furthermore, local “ownership” of these projects is often lacking. For this reason, and in the context of several high-level meetings to promote aid effectiveness (the most important ones in Paris, Accra, and Busan), donors have agreed they should leave more ownership over aid and over development processes in general to recipient countries themselves. This holds, in particular, for technical assistance programs that aim to foster capacity in developing countries. One of the concrete targets agreed in the “Paris Declaration” is that donors commit themselves to coordinate technical assistance programs under the leadership of the recipient country (OECD/DAC, 2005).

Yet, a recent study of the implementation of this agenda finds that there is still a large gap between what donors preach and what they practice (Keijzer, 2013). Most capacity development programs are still supply driven. In addition, they tend to focus on the design phase of the policy cycle and assume that implementation automatically comes forward, thus neglecting the politics involved. The question is why it is so hard to change these practices. An important reason on the supply (donor) side appears to be that those who carry out the technical assistance programs benefit from these programs. Other authors point to spending pressures of donors. As long as capacity development is linked to funding, its effectiveness is limited (Booth, 2013).

However, recipient countries also prove to have an interest in unsuccessful capacity development projects. Civil servants participating in those projects get paid for attendance to seminars, or may travel abroad for training workshops (Armstrong, 2013). This serves to finance patronage relationships. In order to continue these projects, recipient countries send out the correct signals in order to convince donors that institutional changes have been made, but in fact no such changes have occurred (Andrews et al., 2012). This has been called “isomorphic mimicry”: countries officially take over the blue prints and externally suggested “best practices,” but these “policy reforms” only exist on article. Actual practice does not change. Countries may then end up in a “capability trap”: while they continue to “reform,” the gap between formal laws and regulations and actual performance widens over time (Andrews et al., 2012).

At the macro level, several authors have suggested that donors should no longer attempt to impose the first best institutional solutions or best practices in developing countries. Instead, they should be looking for “good enough governance” (Grindle, 2004), “just enough governance” (Levy & Fukuyama, 2010), “second-best institutions” (Rodrik, 2008), or the “best fit” (Booth, 2012).

Recently, these ideas have also been applied to the design of micro level interventions for capacity development and institutional reform. A study by ODI (Overseas Development Institute) of four donor efforts at improving governance in fragile states concludes that donors can help bridging the gap between formal systems and actual performance. The authors recommend that donors should try to exploit political commitment by looking for changes with tangible political payoffs. They also find that it may be much better to try and get things working with existing legal mandates instead of trying to change these legal structures. And yet another lesson is that donors can facilitate problem solving and local collective action by bearing the transaction costs (Tavakoli et al., 2013).

Hyden (2013) explores the reasons for the failure of many public sector reforms in Africa and concludes that, too often, external consultants have ignored the existing administrative culture. They have expected quick and broad changes. Yet, reform efforts that are “comprehensive rather than piecemeal [and] standardized rather than adapted to local circumstances” are unlikely to achieve their objectives (Hyden, 2013, p. 930). In the same vein, Pessali (2011) argues that attempts at policy transplantations should be accompanied by broad consultations with all stakeholders in order to not only adjust them to local situations but also to redesign and improve them in order to achieve better outcomes.

Booth (2013) examines three “semi-autonomous enabling organizations.” They attempt to improve the working of government institutions by directly working with them without having a formal relationship with big bilateral or multilateral aid agencies. They can act more as advisors than as aid providers and are therefore more likely to facilitate institutional change. A survey among the involved officers themselves reveals that four features were particularly important for the success of their work (Booth, 2013, pp. 7–8):

1. Not having a preestablished influencing agenda
2. Finding solutions to problems and facilitating change
3. Flexible performance monitoring that reward learning and adjustment
4. Being answerable to local stakeholders

Andrews et al. (2012) come to similar conclusions. They argue that the above mentioned capability traps are the result of the mainstream approach to capacity development. Most interventions begin with external solutions that are seen as “best practices.” In addition, they assume a linear learning process, imply tight monitoring of inputs and of the predesigned plan, and assume a top-down process in which implementation is automatic. Instead, the authors suggest a problem driven iterative approach (PDIA), with the following components: the interventions should be based on local problem definitions, they should allow for incremental changes and experimentation, they should provide an environment conducive for interactive learning and iterative feedback, and they should engage many different agents in order for reforms to be “viable, legitimate, and relevant” (Andrews et al., 2012, p. 2).

Armstrong (2013) argues that government capacity development is a wicked problem. This implies, among other things, that there are different problem definitions and that there is not one unique solution. He argues that capacity development can only be successful if it is based on codiagnosing, codesigning, coacting, and colearning. However, given that capacity development is not a linear process, these four features should not be seen as subsequent phases in a process. Codiagnosing continues during codesigning and coacting, and colearning underpins all phases (Armstrong, 2013, p. 166).

Codiagnosing means that external experts do not come with normative standards from outside (“best practices”), but begin by investigating what the problem is. Given that there may be different problem definitions, an important aspect is also to examine the “change context.” An analytical method for this is carrying out a stakeholder analysis, identifying who may win and who may lose from change or from its absence. The concept of colearning is related to action learning (Revans, 1979, cited in Armstrong, 2013, p. 117) with the following operational definition: Learning = Programmed knowledge + Questioning + Reflection + Implementation. Based on this, the four principles of colearning are: bringing in technical and adaptive learning expertise, codiagnosing, codesigning, and coacting. Codesigning implies that participants in the project search for incremental changes. Grand designs should be avoided as codesigning is an iterative process. When objectives need to be formulated, this should preferably be done in terms of changes in behavior and in relationships. Coacting is an essential part of the process, because without action no learning can take place. Leaders are important in this process, but they should “skilfully reduce expectations of a top-down magical solution” to a problem (Armstrong, 2013, p. 168). Solutions should originate bottom-up, and in the form of incremental changes, enabled by a network of leaders.

The role of the external expert in the whole process varies from being coordinator, catalyst, observer, climate setter, communication enabler, and learning coach (Armstrong, 2013, p. 174). Colearning and coacting can only come about when the projects’ contracting relationships and accountability mechanisms allow for the possibility of flexibility, experimenting, and eventually, also failures. This often implies that donors’ accountability systems must change.

From this overview of recommendations for successful capacity development, we can deduce a number of success conditions that appear to be important. These include:

1. No “first best” solutions or “best practices” from outside, but broad local stakeholder involvement in problem diagnosis.
2. No grand designs but incremental changes, getting things working with existing legal mandates instead of trying to change legal structures.
3. Formulate objectives in terms of changes in behavior and relationships.
4. The use of technical bodies at arm’s length of the donor.
5. The external actor is facilitator and coach, but is not involved in decision-making.
6. The donor money can bear the transaction costs of collective action but should not provide excessive material resources in the form of attendance fees or travel reimbursements.
7. Adaptive learning as central feature of change processes.
8. Local stakeholders should be involved in monitoring.

The presence of these eight conditions for success will be examined for the PAANEEAC program.

## Origin of PAANEEAC and Methodology

Donors have promoted EIA in Africa for decades. Major donors such as the Word Bank, the USA, Canada, Sweden, Norway, Denmark, and the Netherlands have required EIA to be undertaken for the development projects they financed. Yet, EIA systems in Central Africa remain weak (Bitondo, Post, & Van Boven, 2013). They lack resources, capacities, transparency, and participation. Project consent decisions are often still made without EIA, and even if EIA is applied, its outcomes are often not enforced. The authorities involved are not yet publicly accountable for their decisions.

In the early 2000s, the concern for sustainable development and good governance has led to initiatives by EIA professionals in African countries to improve legislation and practice of the national EIA systems and to increase their ownership. EIA professionals work in EIA administrations, other government sectors, academics, environmental or social NGOs, or with private investors and consulting firms. In many African countries, they have formed national EIA associations, which are cooperating through subregional nodes. An African network of EIA associations emerged, and the Central African node is SEEAC. This process was partly driven by the availability of Dutch donor funding through NCEA, and NCEA was an informal partner in these networks.^3^

In order to be able to receive more extensive funding from the Netherlands, SEEAC began to develop a proposal for a program to support the Central African nodes, with the overall aim to promote EIA as a tool for sustainable development. During these preparations, and due to the fact that the Dutch Ministry of Foreign Affairs required country-specific approaches, the NCEA was asked to develop the methodology for EIA mapping exercises at the national level. EIA mapping implies that participants jointly answer extensive sets of questions about their national system of EIA and about decision-making about development projects in their country. Questions are based on experience with EIA systems strengthening from all over the world, without imposing certain best practices as being applicable in all countries. In 2005 and 2006, the initiators of the NAs in a number of Central African countries invited a larger group of stakeholders to undertake such a national EIA mapping exercise. NCEA facilitated these exercises. These mappings catered to a joint diagnosis among stakeholders of their joint challenge, and led to a debate about potentially effective actions at country level. It was found that the similarities between countries enabled a joint approach, while allowing for country-specific actions plans. This was going to be the basis for the PAANEEAC program, which was approved by the Ministry of Foreign Affairs in 2006. The Ministry asked NCEA to administer the program and to provide technical assistance.

After approval of the PAANEEAC program, the preparation of the contracts took several years. The individual program contracts with SEEAC and with the NAs of Burundi, Cameroon, Congo Brazzaville, Central African Republic, and Rwanda were signed in 2008 and 2009. The NAs of some other countries did not demonstrate sufficient management capacity to qualify for the program.

The program is a mix of core funding, technical assistance, and activity seed money. The associations received just enough core funding to compensate for the cost of housing, basic equipment, and the salary of one staff member. The amount of money spent on core funding and on financing of activities was about equal to the amount spent on technical assistance and coaching. NAs and SEEAC were obliged to prepare annual action plans, annual reports, and project proposals to acquire core funding and small grants for the next year or activity. NCEA examined these plans, reports, and proposals, comparing them with contracts and performance before disbursing funds. This approach allowed for proper financial management of the program, but also facilitated development of project management capacity within the NAs. The intervention logic of PAANEEAC is summarized in Table 1.

**Table 1.** Intervention logic of PAANEEAC according to SEEAC’s original project proposal

Impact	NAs contribute to EIA as tool for good governance, poverty reduction and sustainable development
Outcome	Platform of professionals; legal and institutional framework; capacity of all actors improved; acknowledgement of the role of EIA in governance;
	Thirteen specific outcomes
Output	Activities by NAs and SEEAC
Input	DGIS: Seed funding
	NCEA: Technical Assistance, Coaching
	SEEAC and NAs: General functioning and implementation of action plans

**Source:** Based on NAs and SEEAC (2006).

The information on PAANEEAC and its results comes from a review of the program by the associations themselves (Bitondo et al., 2013), NCEA’s final program report (NCEA, 2014a) as well as by an evaluation carried out by one of the authors who had not been involved in the program (NCEA, 2014b). For this evaluation, the following activities were undertaken between February 2013 and October 2013:

1. Dozens of PAANEEAC-related documents were reviewed, including the original program proposal, annual plans, and annual reports made between 2008 and 2013 by each of the NAs and by SEEAC and project plans and reports of activities, reports of the annual conferences at Central African level, financial reports, donor reports, and EIA mapping reports by the NCEA.
2. 75 interviews were conducted in all five PAANEEAC countries, with NA board members, civil servants in the environment ministries and in line ministries, environmental consultancies, academics, representatives of the business community, politicians, members of the press and of environmental NGOs, other donor organizations present in these countries, and with respondents active at the subregional level: the Central African union CEEAC and SEEAC. Some of the interviews were done in small groups.
3. The evaluator was observer in coaching sessions between NCEA and NAs, in EIA mapping sessions in Congo, Cameroun and Central African Republic, and at the closing meeting at Douala, October 2013 (where evaluation results were presented and discussed).
4. EIA mapping sessions in three countries were used to discuss—after the EIA mapping—the effectiveness of PAANEEAC in a larger group (20–30); some of the 75 interview respondents were also present at these EIA mappings and meetings.

## The PAANEEAC Activities

The 2005–2006 EIA mapping exercises were interactive multistakeholder meetings where a diagnosis was made of the national EIA system and associated processes of governance. They formed the basis of national action plans which, in turn, led to requests for financing. NCEA advisors (usually one) were present at these meetings as cofacilitators; the main facilitator was a Central African EIA expert, who was primarily trained for this role by the NCEA. The EIA mapping method provided for foreign examples, but these were used for inspiration, not for being copied like a blueprint. During these mapping exercises, officers from the highest management level of several government sectors spoke freely about their situation in the presence of EIA stakeholders. This led to a common definition of feasible and timely actions that were likely to have impact on the quality of the EIA system.

Within the general objectives of PAANEEAC, NAs and SEEAC were at liberty to propose activities, seeing to an equal distribution of available resources between them. Some activities were implemented in one or several countries; others were combined activities with all countries. The NAs and SEEAC were in the lead of the implementation of activities, whereas NCEA offered its technical expertise. The NAs are made up of “neutral” or “apolitical” EIA professionals, including members who work in the EIA administrations. In each country, joint projects were implemented by the NAs and their EIA administrations—this time in their formal capacity—together. These practical projects enabled to make direct adjustments to the EIA system. Many other line ministries were indirectly also joining in this process, since their servants are members of the NAs. Civil servants often also participated in activities at subregional level. In sum, the program enabled platforms for interaction (formal and informal, and at national and subregional level) that had not existed before. At these platforms, exchange of ideas was mixed with inspiration from international experts. In the final year of PAANEEAC, members of these associations spent about 10 man-years of voluntary time in activities of these platforms.

The small grants program financed base funding and activities. The activities proposed and financed with seed money for travel and meeting cost included amongst other things: the EIA mappings themselves, specific thematic trainings and seminars, joint projects between NAs and EIA administrations, for example, to develop EIA legislation and data management systems, and annual subregional meetings where general assemblies were combined with thematic seminars.

Initially, the management of the SEEAC and the NAs and their accounting of PAANEEAC subsidies to NCEA were weak. Over the lifetime of PAANEEAC, NCEA dedicated more than half of its time to coaching the staff of the NAs and SEEAC in how to carry out the management in a professional way. An important element was individual coaching of leaders of the NAs. Because financing was conditional on a sound application of the planning and accounting cycle, the NAs had an incentive to improve. The planning cycles of each NA were closely linked. Annual action plans were discussed and coordinated in subregional annual meetings. This peer process not only improved the general quality of strategy and activities, but also made it easier for the NCEA to monitor and approve.

## Program Outcomes

At the end of PAANEEAC, in 2013, the five participating countries did a second EIA mapping. Although many challenges are remaining, there were also clear signs of progress, and PAANEEAC can claim a contribution to this progress. PAANEEAC has induced an acceleration of improvement of legislation and practice of EIA. The number of paying members of NAs has increased from virtually zero in 2008 to about 150 in 2013. For the first time, there was constructive dialogue between stakeholders of EIA and government planners, primarily the EIA administration itself. In most countries, however, participation of certain stakeholder groups is still weak, notably EIA professionals working at local authorities, economic investors, and—to a lesser extent—EIA consultancy firms and environmental and social NGOs.

Early in the program, in the discussion about subregional harmonization of national legislation with a view to coordinated planning and decision-making, and to creating a regional sustainable level playing field for investors, an administrative equivalent of SEEAC proved to be lacking. The establishment of a network of EIA administrations in Central Africa therefore became part of the PAANEEAC agenda. At the end of the program, the Economic Community of Central African States (ECCAS) offered to host the network giving it more credibility, influence, and sustainability. Upon request of the ECCAS, SEEAC and the NCEA will continue their technical support to the setting up of this network.

Concrete PAANEEAC program outcomes include EIA training centres, more well-trained EIA professionals, and EIA documentation centres. In addition, the program is widely believed to have contributed to improved legislation and better practice of EIA. The number of implemented EIAs in the region is increasing and many interviewed stakeholders believe that the general quality of EIA processes improves. There are indications that some EIAs have led to different—presumably more sustainable—development projects, but this is not easy to assess.

Most NAs have achieved an adequate level of internal financial management. In addition, the NAs, with NCEA as their main technical advisor, have produced some outcomes of potentially international significance. This includes a study on the financial basis of EIA systems around the world and in Central Africa (NCEA, 2013), and a joint analysis of the evolution of EIA in their countries (Bitondo et al., 2013).

Some other donors supported some NAs and SEEAC in the PAANEEAC period. This related to activities in full synergy with the NA’s action plans that had resulted from PAANEAAC. This contributed to PAANEEAC’s objective to help the NAs and SEEAC become financially independent from base funding, able to acquire projects fitting their “mission.” After PAANEEAC, NAs will to some extent remain dependent on external funding as do similar organizations in Western countries. Yet, other envisaged outcomes have not been achieved, such as the publication of key decisions, and the putting in place of environmental norms and standards. EIAs are still often not undertaken, or not fully undertaken, due to a lack of resources for public tasks. Yet, respondents indicate that they now personally have a better understanding of what is necessary to improve EIA and governance with a view to sustainable development; and since they share these views in influential networks, they are also optimistic about chances of further improvement. The shortcomings of governance (including corruption) have been well denounced in meetings and groups. Relationships with politicians and journalists were part of these discussions, as a transition toward better governance needs to be enabled by politicians, and politicians may be sensitive to the media.

Sustainability of the EIA associations as effective platforms after PAANEEAC depends on their ability to attract funds without themselves becoming involved in individual EIAs, which would lead to losing their neutral position and would bring the risk of the association getting into competition with its members. Present performance is based on a paid staff member, so the NAs will need to receive member fees and/or contributions from EIA administrations and international donors. Some leading NA members have the intention to continue their subregional peer process in the planning and reporting cycle, not only to improve their performance but also to remain attractive to donors. It remains to be seen whether all NAs will succeed in attracting sufficient funds to continue performance at current levels.

## Presence of Success Conditions

### No “First Best” Solutions or “Best Practices” from Outside, but Broad Local Stakeholder Involvement in Problem Diagnosis

At the start of the program, the EIA mapping exercises, which involved a wide range of local stakeholders, played an important role in identifying problems and feasible solutions. The NCEA and SEEAC jointly facilitated these exercises but did not impose its outcomes. Also after this start, PAANEEAC was built around problems that were jointly defined by its local stakeholders united in the NAs and in SEEAC. Where NAs looked for solutions, approaches, and actions that were appropriate for their situation, NCEA shared examples and experiences from countries all over the world.

The core of the local stakeholder network was—and still is—formed by those EIA professionals whose daily business involves EIA: the EIA administration, EIA experts in other administrations, environmental consultants and aspirant consultants, and academics. The stakeholders of sustainable economic development are more diverse than that, but the NAs have the potential to connect them all. They are extending their network as needed, for instance with journalists. The program proposal was defined by that core network, whilst action plans were based on joint EIA mappings, where dozens of stakeholders participated.

However, in order to increase EIA application and the quality of government services in the EIA systems, in particular in enforcement of license conditions, the NAs will need to expand their networks and should try to raise awareness of the general public about better governance through EIA. They also need to connect to other, more influential stakeholders, in particular economic investors who have an interest in a sustainable level playing field. A broader network will probably lead to more effective improvements in EIA decision-making procedures, in enforcement of decisions, and regional harmonization.

### No Grand Designs but Incremental Changes, Getting Things Working with Existing Legal Mandates Instead of Trying to Change Legal Structures

PAANEEAC activities and projects aimed at changes in the EIA system were not based on grand designs but were of an incremental nature and seen as feasible within local political contexts. One of the four specific PAANEEAC objectives was to improve the legal, regulatory, and organizational framework for EIA, but it is important to note that in most countries, the basic legal institutions and capacities were already in place. It has since long been the view of important donors (primarily banks like the World Bank) that countries should develop their own capacity of making good judgments about acceptable development projects, and that this should be done in a participative and transparent way. To this end, such donors have, with a peak in the 1990s, stimulated the development of legal systems for EIA. NAs have looked for incremental improvements of practice and law by analyzing strengths and weaknesses of the system, and identifying feasible opportunities for improvement. They found that weak points were not only related to practice but are also often still of legal nature (e.g., lack of realistic environmental standards and lack of workable procedures). Some PAANEEAC activities were aimed at wider discussion about such needs and looking for ways to connect to the public and to politics (e.g., by inviting journalists to work together with the NAs).

### Formulate Objectives in Terms of Changes in Behavior and Relationships

Some of PAANEEAC’s objectives were explicitly defined as changes in behavior and relationships, for example, “improved coordination of EIA professionals” and “broad stakeholder involvement in decision making” (NAs & SEEAC, 2006). In practice, behavioral goals were even more dominant. Whereas many participants of PAANEEAC were primarily driven by a concern for sustainable development, it was always clear that a platform for debate—national and regional—was the prime outcome. The other foreseen outcomes were spinoffs of the platform. All participants in PAANEEAC activities and meetings were volunteers. Strikingly, over 70 respondents from five countries all explained their enthusiasm in a similar way: they were concerned with good governance with a view to sustainable development, and they believed improving EIA to be a good and lucrative start.^4^

The NAs, as platform for debate about the quality of governance that is, the way in which the government makes its decisions were conducive to changes in behavior and relationships. The different stakeholders did not see each other anymore as opponents or business partners only, but also as strategic partners that all depend on better governance for their joint interest: sustainable development. Cooperation between such contrasting partners was often discussed during PAANEEAC sessions. The NAs explicitly looked for funds that would not compete with the interests of any of their members; for example, the NAs could not be hired for services in the EIA procedure.

The achieved changes in behavior include the self-governance of EIA professionals, their relationship with government in the policy process, and the fact they have taken responsibility for financial autonomy. A growing and credible network with technical capacities to draft project proposals and administer projects has resulted. NAs are now increasingly working with other stakeholders, in particular national administrations in charge of environmental assessment and the ECCAS.

### The Use of Technical Bodies at Arm’s Length of the Donor

The NCEA acted as a technical body on behalf of the donor, the Ministry of Foreign Affairs. It is plausible that the existence of the NCEA as technical body with room for maneuver to enable, stimulate, and be patient enabled a favorable program context. From the beginning, the NCEA believed in the possible benefits of a regional approach combined with small grants, and was of the opinion that it would be interesting for NCEA itself to undertake such a program to see if it really works.

At the same time, the NCEA could afford to be patient. NCEA did not want to sign program contracts before an NA met certain requirements, even though the budget was already available. NCEA even rejected some NAs, and the contracts with two NAs were terminated during the program because they no longer complied with the requirements. PAANEEAC offered EIA professionals an opportunity for professional learning, without paying for their time, and left responsibility for good governance and action primarily with the NAs. The NCEA had always indicated that five years (which due to a slow start became six) would have to be enough for the NAs to become autonomous. NAs at first reacted slowly; it took them a long time to organize their own governance before PAANEEAC could start. In this period, the NCEA itself spent some time on communication, and the promise of a program remained “in the air”. Next, it took several years for the program to get vibrant. The final years were of high activity, with many voluntary man-years spent. NA members increasingly seemed to believe in their own learning and improvement process, and in their ability to continue a high level of self-governance after the ending of PAANEEAC.

### The External Actor is Facilitator and Coach, but is not involved in Decision-making

The NCEA enabled these processes but did not interfere with decision-making. The NCEA technical advisors spent more than half of their time on coaching the NAs and SEEAC in management, and in carrying out the planning and control cycle with a view to professionalization of the NAs. The NCEA advisors provided comments to annual plans and reports, and the NAs and SEEAC highly appreciated their reflections. Yet, the initiative for activities was in the hands of the NAs and SEEAC. There was also a strong synergy with technical EIA assistance supplied. The NCEA advisors developed the EIA mapping method and facilitated its implementation. They provided technical advice on projects and activities by the NAs, and they developed and carried out a training of trainers. NCEA also delivered the trainers for the more technical workshop and training activities, in case such expertise was not available in the countries.

### The Donor Money Can Bear the Transaction Costs of Collective Action but Should Not Provide Excessive Material Resources in the Form of Attendance Fees or Travel Reimbursements

PAANEEAC provided budget for national seminars and, once a year, a general assembly of SEEAC’s members combined with an international seminar alternatingly in one of the participating Central African countries. The small grants were merely covering travel and meeting expenses, and did not cover fees or time input of participants. In this way, the donor money was bearing the transaction costs of establishing national and subregional platforms. NCEA deliberately chose this approach to ensure that participants were primarily driven by the program objectives and indeed, PAANEEAC mobilized considerable voluntary time of hundreds of EIA professionals.

### Adaptive Learning as Central Feature of Change Processes

The program structure was robust enough to enable continuous learning (see Table 1). It was clear enough to keep focus on desired change. The annual planning and reporting cycle allowed for taking up new activities or cancelling interventions originally considered as suitable or necessary, when circumstances so dictated or in case new priorities came up. In this way, the PAANEEAC program moved along with the contextual agenda (as opposed to following an imposed donor’s agenda).

### Local Stakeholders Should be Involved in Monitoring

PAANEEAC’s active stakeholders jointly monitored progress toward desired change and adapted their joint and separate actions accordingly. They did this primarily through their annual planning and reporting systems, which helped in promoting local accountability. The two multistakeholder EIA mappings conducted in 2006 and in 2013 in all program countries also played a role in progress monitoring.

Yet, there was another tier of accountability as well, namely accountability to the donor through NCEA. At the start of the program, NCEA verified, with the Ministry, whether the PAANEEAC program proposal was in line with the Ministry’s overall objectives. During the course of the program, the approved proposal was used as reference for coaching the African partners and for decision-making about funding proposals. Before approving activities, NCEA checked the justification and quality of proposals against the original program objectives and the joint planning process. Afterward, NCEA required the NAs and SEEAC to account for the implementation of the financed activities. Very often, NCEA had to (initially) reject proposals and financial reports or ask for amendments. This external accountability has been important for improving the NAs’ capacities for conducting sound planning and management of their operations. They had a clear incentive for improving their performance, and the external accountability combined with technical assistance and coaching from NCEA staff, helped them to do so. In practice, this meant that the program influenced self-organization toward a governance that is better able to deal with complexity (e.g., Edelenbos & Eshuis, 2009).

## Conclusion

The aim of this article was to assess to what extent PAANEEAC, a capacity development program to improve national EIA systems in Central African countries, was successful, and which factors contributed to this success. The starting point was that most donor projects for capacity development have had very little impact. This article reviewed the emerging literature on why this is the case. From this literature we derived conditions for success, which include:

1. No “first best” solutions or “best practices” from outside, but broad local stakeholder involvement in problem diagnosis.
2. No grand designs but incremental changes, getting things working with existing legal mandates instead of trying to change legal structures.
3. Formulate objectives in terms of changes in behavior and relationships.
4. The use of technical bodies at arm’s length of the donor.
5. The external actor is facilitator and coach, but is not involved in decision-making.
6. The donor money can bear the transaction costs of collective action but should not provide excessive material resources in the form of attendance fees or travel reimbursements.
7. Adaptive learning as central feature of change processes.
8. Local stakeholders should be involved in monitoring.

The article shows that PAANEEAC has put many of these conditions in practice. The program has been based on local problem definitions, avoided grand designs, and focused on changes in behavior and relationships. In addition, the NCEA has played the role of facilitator and coach, as a technical body, while the donor money was mainly used for financing the transaction costs of network meetings. The annual planning and reporting cycle allowed for adaptation and learning, and project activities were monitored by local stakeholders when preparing their annual reports.

Yet, the article also shows two instances in which PAANEEAC deviated partially from these success conditions. First, although the NAs brought together a wide range of EIA stakeholders, participation from environmental NGOs, in particular from EIA professionals working with private investors interested in a sustainable level playing field can still be improved in most countries.^5^ This also affects the success of the program itself. If and to the extent NAs succeed in expanding their membership, this will probably enhance the chances of improving EIA systems as well. Second, although local stakeholders played an important role in the monitoring of activities, there was also an important accountability outward and upward, to the NCEA and through it, to the Ministry of Foreign Affairs and the Dutch tax payers. This deviation, however, proved beneficial for the consolidation of the NAs. The combination of financial incentives and technical assistance provided by NCEA led to great improvements in the planning and management capacities of the NAs. This importance of accountability upward and outward, in addition to local accountability, was not brought up by the literature. Yet, and perhaps somewhat paradoxically, it proved crucial for creating capable and autonomous organizations. It may be relevant for other external interventions as well.

The main PAANEEAC outcome proved to be the creation of national and subregional platforms in which stakeholders meet with the common objective of improving EIA systems. PAANEEAC mobilized considerable voluntary time of hundreds of EIA professionals. It has led to the building of new relations between stakeholders around a common theme. An ingredient for this self-organization may be the presence of fertile soil for a common discourse, with a clear and uncontested goal—in this case sustainable development. However, the aid program has certainly contributed as well to the self-organization of EIA professionals—not a minor achievement. PAANEEAC has served the process of looking for piecemeal interventions in legislation or practice that best fit the local situation. A certain capacity gap is in many situations considered constructive: many respondents indicated that a country first needs ambitious procedural legislation before a practice slowly can emerge. The robustness and combined diverse influence of these social networks suggest that they may continue to have that effect. The probability of achieving more major, nonlinear, improvements of the EIA and decision-making systems has increased. This may enhance the chances of sustainable development in the countries.

Yet there are major uncertainties for the future. The NAs will continue to depend on external funding as do similar organizations in Western countries. Prospects for attracting funding vary from country to country. In addition, the NAs have no formal power and depend on a favorable context to sustain their performance. That context is now improving, viewing the fact that several NAs and SEEAC have now been formally recognized by government or international government organizations as technical partners in the field of EIA. The social networks builtup may fall apart if members lose their belief that they can make a difference.

All in all, the PAANEEAC program had measurable, albeit as yet still modest, effects on the evolution of the EIA systems. Although the program’s first impact was on relations within the community of EIA professionals and between this community and governments, in respondents’ views there were also clear effects on EIA practice and legislation. The fact that most of the conditions for success were met has probably played an important role in this performance. The PAANEEAC experience also shows that capacity building takes a long time and requires dedicated efforts. The preparation period for PAANEEAC was long and the combined cost of management coaching and technical assistance was of the same order of magnitude as the small grants themselves. An approach like PAANEEAC, aiming at self-organization and local ownership of change processes, requires patience.

## Notes

1. Retrieved from November 13, 2013, from http://www.oecd.org/dac/capacitydevelopment.htm

2. Retrieved from www.SEEACOnline.org

3. Later on, NCEA developed into a formal partner.

4. Yet, for different member categories there were additional specific drivers. Consultants, for example, hoped that increased EIA application would lead to more consultancy work, and EIA agency staff hoped to increase their prestige and importance.

5. Western private investors proved to be interested in a fair market for sustainable investments in Africa (e.g., Porter & Kramer, 2011; Van Tilburg, Van Tulder, Francken, & Da Rosa, 2012, p. 28).
